# In Vitro Rumen Fermentation Characteristics, Estimated Utilizable Crude Protein and Metabolizable Energy Values of Grass Silages, Concentrate Feeds and Their Mixtures

**DOI:** 10.3390/ani13172695

**Published:** 2023-08-23

**Authors:** X Muqier, Margrete Eknæs, Egil Prestløkken, Rasmus Bovbjerg Jensen, Katrine Sømliøy Eikanger, Inger Johanne Karlengen, Gisken Trøan, Stine Gregersen Vhile, Alemayehu Kidane

**Affiliations:** 1 Department of Animal and Aquacultural Sciences, Faculty of Biosciences, Norwegian University of Life Sciences (NMBU), P.O. Box 5003, N-1432 Ås, Norway; 2 Norgesfôr AS, Akershusstranda 27, N-0150 Oslo, Norway; inger.johanne.karlengen@norgesfor.no

**Keywords:** gas production, ruminant feed, energy value, short-chain fatty acids

## Abstract

**Simple Summary:**

We report the in vitro rumen fermentation characteristics, utilizable crude protein, and energy values of four different formulations of concentrate feeds, three contrasting qualities of grass silages, and mixtures thereof (45% concentrate, 55% silage, dry weight basis) using the ANKOM RF wireless gas production system. The concentrates were two pelleted diets (control concentrate for dairy cows and alkaline concentrate with ammoniated barley) and two mash forms (concentrate with ingredients of the alkaline diet prior to alkalization and concentrate with basal ingredients as in the alkaline diet with feed-grade urea). The grass silages were early cut, late cut, and a 1:1 mix thereof. For mixed diets, no interaction effects of the concentrate feeds by silage quality were observed for the tested parameters. For concentrates, the pelleted diets were higher in in vitro dry matter digestibility and molar proportion of propionate. The alkaline pelleted concentrate produced a higher utilizable crude protein value than others. For silages, estimated protein and energy values, total short-chain fatty acids, and molar proportions of propionate and branched-chain fatty acids decreased with increasing stage of maturity. Results for silages and their mixtures with concentrates highlight the importance of silage quality in dietary energy and protein supply for ruminants.

**Abstract:**

Four formulations of concentrate feeds, three contrasting qualities of grass silages, and mixtures of the silages (55%) and concentrates (45%, dry weight) were tested for in vitro fermentation kinetics, in vitro dry matter degradation (IVDMD), utilizable crude protein (uCP), and metabolizable energy (ME) values. The concentrates were pelleted control concentrate for dairy cows (CONT-P); pelleted alkaline concentrate with ammoniated cereal grains (ALKA-P); mash form concentrate with ALKA-P main ingredients but with feed-grade urea and barley replacing ammoniated cereal grain (UREA-M); and mash form of ALKA-P ingredients prior to alkalization (ALKA-M). The grass silages were early cut, late cut, and a mixture (1:1) of early and late cut. The objectives were to test if the feeds differed in the tested parameters within each feed category and assess the modulatory effect of concentrate feeds on the grass silage fermentation characteristics in the mixed diets. No interaction effects of the concentrate feeds by silage quality were observed for the tested parameters in the mixed diets. For concentrates, the pelleted diets were higher (*p* < 0.05) in IVDMD and molar proportion of propionate but lower in butyrate. The ALKA-P produced the highest estimated uCP (*p* < 0.01). For silages, uCP, ME, total short-chain fatty acids (VFAs), and molar proportions of propionate and branched-chain VFAs decreased (*p* < 0.05) with increasing stage of maturity. In conclusion, the ALKA-P could match the CONT-P in uCP and ME values and fermentation characteristics. Results for silages and their mixtures with concentrates highlight the importance of silage quality in dietary energy and protein supply for ruminants.

## 1. Introduction

A major part of Norwegian plant production is best suited for livestock [1] with about one-third of the 3% arable land used for grain production. Furthermore, a large portion of this grain production (≥85%) is used as animal feed due to stringent quality requirements in place for food grains [2]. On the contrary, local production of protein ingredients is limited due to climatic challenges, leading to the importation of a large portion of these ingredients in concentrate feeds for production animals [3]. Efforts to increase or sustain the current level of (e.g., milk) production based on local resources (i.e., both roughages and concentrate feeds) with minimal recourse to imported ingredients has necessitated improving both energy and, most importantly, the protein value of these feed resources.

For roughages, improving the protein and energy value (e.g., grass silage commonly used in the Scandinavian countries) through either manipulating the stage of maturity of the crop or altering the silage fermentation pattern is not sufficient alone to meet animal requirements. As such, these feeds are augmented with different levels and qualities of concentrate feeds to achieve a given level of production. For concentrate feeds, cereal grain ingredients are high in starch (i.e., energy source), but they are low in protein content. Thus, maintaining the current level of production based on these local resources inevitably requires novel feed resources, new technologies, and treatment methods. To this end, alkaline concentrate feed prepared by mixing locally produced cereal grains with Home n’ Dry pellets (i.e., Alkagrain^®^, Alkasystems; Dugdale Nutrition, Lancashire, UK) is becoming a common ruminant feed in Europe [4]. Here, mixing the Home n’ Dry pellets with cereal grains releases ammonia, which binds to the grains in the form of ammonium salts, resulting in alkalization. This alkalization process, among other things, increases the level of crude protein as non-protein nitrogen [5] for ruminants and is hypothesized to offer increased buffering capacity due to its relatively higher pH (8.0 to 9.0). This would create a rumen environment conducive to dietary fiber degradation [6] with an opportunity to increase the inclusion level of local grains in concentrate feeds to high-producing animals.

Furthermore, even though ingredient composition is the primary driver of the nutritive value of concentrates, processing can substantially alter the nutritive value of feeds for cattle [7,8]. For instance, pelleting increases starch gelatinization and hence can improve diet fermentation [8,9,10] and dietary energy availability. Similarly, heat treatment of protein ingredients increases the duodenal flow of amino acids [11,12]. On the other hand, the use of silage additives [13], the degree of wilting at ensiling [14], and most commonly, modulation of the stage of crop maturity at cutting [15] are some of the management tools to alter the nutritive value of grass silages. Here, we assessed the in vitro rumen fermentation kinetics, estimated the metabolizable energy (ME) and utilizable crude protein (uCP) values of different formulations of concentrate feeds, contrasting qualities of grass silages, and mixtures of the concentrate feeds and grass silages (assuming a hypothetical dairy cow ration) using the ANKOM RF wireless gas production system (ANKOM Technology, Macedon, NY, USA). We hypothesized that the different formulations of concentrate feeds (i.e., varying in ingredient composition and pelleting treatment) and the contrasting qualities of grass silages modulated through the stage of maturity at cutting would differ in their in vitro fermentation characteristics, estimated ME, and uCP values. We further hypothesized that the poor quality late cut grass silage in the mixed diets would benefit more in fermentation parameters from the modulatory effects of concentrate feeds as observed through the interaction effect of grass silage quality and concentrate feed type. Lastly, we hypothesized that an alkaline grain diet would counteract the drop in the endpoint pH of the incubated media through its anticipated higher buffering capacity—indicative of in vivo feeding conditions.

## 2. Materials and Methods

### 2.1. Experimental Design and Description of Feed Materials

This experiment involved the characterization of chemical composition and estimation of uCP (g/kg DM) and ME (MJ/kg DM) values of four concentrates, three grass silage qualities, and mixtures of the concentrate feeds and grass silages.

The four concentrates were: (1)—pelleted control soy-based concentrate formulated for high-yielding dairy cows (CONT-P); (2)—pelleted alkaline concentrate formulated with ammoniated cereal grains (ALKA-P); (3)—a mash form of the ingredients as in ALKA-P but with feed-grade urea and barley replacing ammoniated cereal grain (UREA-M); and (4)—a mash form of the ingredients of ALKA-P prior to alkalization (ALKA-M). The alkalization process of the ALKA-P involved blending concentrate feed ingredients, composed largely of barley as cereal grain, with Home n’ Dry^®^ (i.e., Alkagrain^®^, Alkasystems; Dugdale Nutrition, Lancashire, UK) pellets with subsequent closed system reaction and release of ammonia into the blend prior to pelleting. The CONT-P was used as a positive control in the dairy cow ration, whereas the UREA-M was included as a negative control to see the benefits of alkalization from ALKA-P, given similar nitrogen from the feed-grade urea. Where Home n’ Dry pellets were mixed with ALKA-P basal ingredients just before the in vitro work (i.e., eliminating further reaction in the feed mixture) the ALKA-M was used to see the effects of the alkalization and pelleting treatment in ALKA-P. All concentrate feeds were formulated to be iso-nitrogenous and iso-energetic.

The selected three grass silages were: (1)—good quality early cut (Ecut); (2)—medium quality (Mcut); and (3)—poor quality late cut (Lcut) with the quality manipulated through cutting age of the grass. The Ecut grass silage was high in dietary crude protein (CP) and low in neutral detergent fiber (NDF), and conversely, the Lcut grass silage was low in dietary CP and high in NDF, whereas the Mcut was a 1:1 mixture (on a dry matter basis) of the Ecut and Lcut.

Furthermore, the grass silages (F) and concentrate (C) feeds were mixed at a ratio of 0.55:0.45 (in respective order; F:C) on a DM basis, creating a hypothetical dairy cow ration. The grass silages (*n* = 3), concentrates (*n* = 4), and their mixtures (*n* = 3 × 4 = 12) produced a total of 19 dietary treatments for in vitro gas production measurements as described below.

### 2.2. Feed Preparation, Chemical Analysis

#### 2.2.1. Feed Preparation, Chemical Analysis and Dry Sieving for Particle Size Distribution

Feeds for in vitro gas production, particle size distribution, and chemical analysis were milled using a cutter mill (Retsch SM 200, Retsch GmbH, Germany) to pass through a 1.0 mm sieve size, whereas the concentrate feeds for starch analysis were milled through a 0.5 mm sieve size. The feed residues recovered from in vitro gas production were homogenized using an IKA basic analytical mill (IKA^©^ A11 basic, Staufen, Germany) in preparation for chemical analysis. Chemical composition of the pure concentrate feeds and the grass silages used is provided in Table 1 (for the mixed diets, the composition can be calculated based on the silage and concentrate feeds’ chemical composition and their proportion in the diet).

The DM content of the samples was determined by drying at 103 °C overnight (ISO 6496; [17]), and the ash content was determined by incinerating the samples at 550 °C (ISO 5984; [18]). The nitrogen (N) content was determined using Method 2001.11 [19] according to Thiex et al. [20] with the Kjeltec 2400/2460 Auto Sampler System (FOSS Analytical, Hilleroed, Denmark) with CP estimated as N × 6.25. Total starch content of the concentrate feeds was analyzed using AACC Method 76–13.01 (Megazyme amyloglucosidase/α-amylase method; [21]) with starch hydrolysis to glucose and determining the concentration of glucose colorimetrically using an RX Daytona+ spectrophotometer (Randox Laboratories Ltd., County Antrim, UK). The NDF was determined with an ANKOM^220^ fiber analyzer (ANKOM Technology, Fairport, NY, USA) using heat-stable amylase followed by combustion at 550 °C and expressed without residual ash (aNDFom), whereas the acid detergent fiber (ADF) was determined according to Method 973.18 [22] and corrected for residual ash (i.e., ADFom). The water-soluble carbohydrate content was determined as described in Randby et al. [23], whereas the residual carbohydrate content was estimated as the DM in the feeds minus the sum of analytical components as described in the Nordic feed evaluation system [24]. The crude fat content of the feeds was analyzed according to the Commission Regulation (EC, No 152/2009) [25] with crude fat extraction in a SoxtecTM 8000 system (FOSS Analytical, Hilleroed, Denmark) using light petroleum as a solvent following Randall [26] modification. The in vitro ruminal fluid samples were analyzed for short-chain volatile fatty acids (VFAs) by gas chromatography (TRACE^TM^ 1300 Gas Chromatograph equipped with Stabilwax—DA column 3 m, 0.53 mm ID, 0.25 μm; Thermo Scientific, MA, USA) and for ammonia nitrogen (NH_3_-N) using Method 2001.11 according to Thiex et al. [20]. For concentrate feeds, the presented feed composition is based on duplicate analysis, whereas for the grass silage the composition is based on quadruplet analysis.

The pH of the feeds was measured in triplicate following the method of Jasaitis et al. [16]. In brief, 0.5 g DM of feed was suspended in 50 mL of distilled deionized water under continuous magnetic stirring at room temperature. The pH was measured using pH 3310 (Xylem Analytics Germany GmbH, D-82362 Weilheim, Germany) after equilibration of the suspended feed for 3 min.

The particle size distribution of the concentrate feeds was evaluated using dry sieving. For this, 5.0 g DM of the concentrate feeds (dried at 103 °C overnight) was sieved using a vibratory sieve shaker (Retsch AS 200 Control, Retsch GmbH, Haan, Germany) in triplicate by installing five screen apertures on top of the receiving plate (i.e., 0.05, 0.10, 0.25, 0.50, and 0.80 mm). This yielded 6 fractions of each feed (5 fractions resting on each screen aperture plus the finest fraction passing through the 0.05 mm aperture to the receiving plate). The sieving was performed at an amplitude of 1.1 mm for a duration of five minutes. The particle size distribution is expressed as the fraction of DM resting on each screen as a percent of the total DM sieved (i.e., % mass fraction), and geometric mean particle size was calculated according to the ASAE [27] method for each feed.

#### 2.2.2. In Vitro Gas Production

The in vitro gas production was carried out using an ANKOM RF Gas Production System (ANKOM Technology; Macedon, NY, USA) with approximately 1.0 g DM of the feeds incubated in triplicate and in two batches using a completely randomized order. Each batch included background bottles (buffer + rumen fluid without added substrate) as blanks and an internal standard feed (a dairy cow diet prepared as a total mixed ration with ingredient and chemical composition provided elsewhere [28], representing a CP of 175 g/kg DM) in triplicate. The samples were incubated in 100 mL of buffered [29] rumen fluid (67 mL buffer solution and 33 mL rumen fluid) for 48 h using 250 mL glass bottles. For this, the rumen fluid was collected about 3 h post-morning feeding from three rumen cannulated cows fed according to the Norfor [30] feeding standard with a diet composition as recently described in Alvarez et al. [31]. The rumen fluid was collected into two pre-warmed thermos flasks and filtered through a nylon cloth (SEFAR NITEX, Sefar AG, Heiden, Switzerland) with a pore size of 200 μm into glass bottles maintained in a 39 °C water bath. The in vitro incubation was performed at 39 °C with continuous gentle shaking on a Stuart SSL3 3D gyro-rocker (Cole-Palmer Ltd., Staffordshire, UK). During incubation, cumulative gas pressure was registered at every 10 min interval by a computer through a wireless communication with individual ANKOM modules. The system was set to release gas at a predetermined headspace pressure (0.75 psi above atmospheric pressure) to avoid pressure buildup in the modules.

After 48 h of incubation, the endpoint pH (pH_48_) of the incubated media was recorded using pH 3310 (Xylem Analytics Germany GmbH, D-82362 Weilheim, Germany), and a 4.75 mL sample from the fluid phase of the incubated medium was preserved with a 0.25 mL of concentrated (98% *v*/*v*) formic acid for VFA and ammonia-N analysis. The remaining in vitro residue was then recovered by filtering through 12 μm pore size nylon bags and washed following the in sacco technique as described in Norfor [30] and dried to determine in vitro DM degradation (IVDMD, %). The IVDMD is expressed as the difference between DM incubated and residual DM after 48 h incubation as a percentage of the DM incubated.

### 2.3. Calculations and Statistical Analysis

The cumulative gas pressure from individual modules was converted into gas volume using the ideal gas law (*n* = (pV)/(RT)), where *n* is moles of gas produced, p is gas pressure in kPa, V is the headspace volume of incubation bottles in L, T is the incubation temperature in K, and R is the gas constant (8.314472 L·kPa·K-1·mol^−1^). The gas produced (GP) was then converted from moles to gas volume using Equation (1):

GP (mL) = *n* × 22,400 mL/mol, (1)

where *n* is as described above, and 22,400 mL/mol is the molar volume of gas at standard temperature (273.15 K) and pressure (101.325 kPa).

The uCP of the dietary treatments was estimated using the method of Edmunds et al. [32]. The method calculates uCP (the sum of feed undegraded protein and microbial protein) by using the ammonia-N concentration (at the end of incubation) in the fluid phase of the blank bottles (NH_3__N_blank, mg) and bottles containing feeds (NH_3__N_sample, mg), and feed nitrogen (N_sample, mg) with the formula as provided in Equation (2):

(2) $$uCP\;(g/\mathrm{kg}\;\mathrm{DM})=\frac{\left(\mathrm{NH3\_N\_blank}+\mathrm{N\_sample}\right)-\left(\mathrm{NH3\_N\_sample}\right)}{\mathrm{Sample}\;\mathrm{weight}\;\left(\mathrm{mg}\;\mathrm{DM}\right)}\times 6.25\times 1000,$$

where 6.25 is used as the conversion factor for N into CP, and 1000 is used for converting the unit from mg/mg DM into in g/kg DM.

The ME content of the dietary treatments was calculated using in vitro gas volume at 24 h and the chemical composition of the feeds according to GfE [33] using Equation (3):

ME (MJ/kg DM) = 7.81 + (0.0756 × GP) − (0.0038 × Ash) + (0.0057 × CP) + (0.0190 × CF) − (0.0083 × ADFom),(3)

where GP is in vitro gas production at 24 h (mL per 200 mg DM) and Ash, CP, CF, and ADFom are expressed in g/kg DM. Furthermore, the net energy for lactation (NE_l_) was calculated using the estimated ME and analyzed ash content of the diets according to Weißbach et al. [34] (Equation (4)):

NE_l_ (MJ/kg DM) = ME × [0.46 + 12.38 × ME/(1000 − ash content)], (4)

Gas production profiles were fitted for individual incubation modules using the model of Groot et al. [35], assuming a single pool fermentation order (Equation (5)):

GP = A/(1 + B^C^/t^C^) (5)

where GP is cumulative gas volume at time t; A is asymptotic gas production (mL/g DM incubated), B is time (h) taken to produce half of the asymptotic gas volume, and C (unitless) is the shape parameter determining the sharpness of the switching characteristic of the gas production profile. For this, Proc NLIN in SAS (SAS for Windows, version 9.4, SAS Institute Inc., Cary, NC, USA) was applied with the MARQUARDT iterative method. The fractional rate of gas production (Rgp) was calculated assuming a fixed linear relationship between substrate fermentation and gas production as described in Groot et al. [35].

Statistical analysis of gas production data from grass silage and concentrate feeds (i.e., gas volume at 12, 24, and 48 h of incubation, parameter estimates, pH_48_, VFAs, uCP, ME, NE_l_, and IVDMD) was carried out using SAS PROC MIXED including dietary treatments and batches as fixed factors and a replicate in a batch as random factor within each feed category. For the concentrate feed-grass silage mixed diets, the model was extended to include the fixed effects of concentrate feed, grass silage quality, and their interaction effects. Differences between treatment means were separated using the Tukey–Kramer test with significance declared at *p* ≲ 0.05 and tendencies discussed at 0.05 < *p* ≲ 0.1. For particle size distribution of the concentrate feeds, the mass fraction resting on each sieve aperture (as % of total DM used) was compared using one-way analysis of variance in SAS and means with standard errors of the mean reported in the Supplementary Materials (Figure S3).

## 3. Results

### 3.1. Feeds and Chemical Composition

The concentrate feeds used were formulated for high-yielding dairy cows as reflected in the nutrient composition. However, numerical differences were noticed among the concentrate feeds for the analyzed chemical components, which could influence their in vitro fermentation parameters. For the grass silages, the differences in chemical composition among the three qualities were graded as planned (Table 1).

### 3.2. In Vitro Gas Production

**Figure 1:**
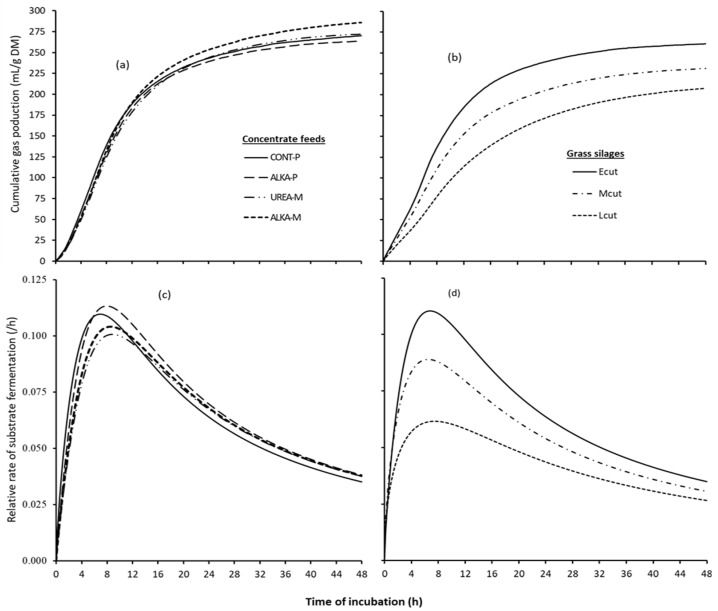
Cumulative gas production of (**a**) concentrate feeds, (**b**) grass silages; rate of substrate fermentation of (**c**) concentrate feeds and (**d**) grass silages. Concentrate feeds: CONT-P = pelleted control concentrate feed, ALKA-P = pelleted alkaline concentrate feed, UREA-M = concentrate feed formulated with feed-grade urea in a mash form, ALKA-M = ALKA-P in a mash form avoiding the ammoniation step. Grass silages: Ecut = early cut, Lcut = late cut, Mcut = a mixture (1:1, on a dry matter basis) of the Ecut and Lcut.

Cumulative gas production profiles and relative rates of substrate fermentation of the pure concentrate feeds and grass silages are presented in Figure 1, whereas data on in vitro gas production and fermentation kinetics of these feeds are presented in Table 2. Mean cumulative gas production did not differ among the four concentrate feeds at 12, 24, and 48 h of incubation, presented as GP_12_, GP_24_, and GP_48_, respectively. Similarly, calculated asymptotic gas volume (A) and R*_pg_* did not differ among the concentrate feeds. However, CONT-P and ALKA-P took a noticeably shorter time (*p* < 0.001) to produce 50% of the asymptotic gas volume (B) compared to UREA-M and ALKA-M.

For the grass silages, there was a significant (*p* < 0.001) effect of silage quality on the above parameters, except for total VFAs and butyrate. As such, gas volume (GP_12_, GP_24_, GP_48,_ and A) and R*_pg_* gradually decreased with increasing stage of maturity of the crop. Conversely, the B parameter estimate was longer for the Lcut, intermediate for the Mcut, and shorter for the Ecut grass silages.

For the mixed feeds, selected gas production and fermentation kinetics data are presented in Table S1 and Figure S1. For gas volumes at different time points (i.e., GP_12_, GP_24_, and GP_48_) and parameter estimates (i.e., A, B, C, and R_pg_) the interaction effects of concentrate feed by grass silage quality were not significant. However, there was a significant effect of grass silage quality (*p* < 0.01) whereby GP_12_, GP_24_, and GP_48_, A, and R_pg_ of the mixed diets decreased with increasing stage of maturity (i.e., Ecut > Mcut > Lcut). This was accompanied by the increasing B parameter estimate (*p* < 0.01) with increasing stage of maturity of grass silage in the mixed diets.

### 3.3. In Vitro DM Digestibility

Calculated IVDMD values are provided in Table 3 for the concentrate feeds and grass silages, whereas for the mixed diets, the values are presented in Figure S2. The IVDMD values were not significantly different between the CONT-P and ALKA-P concentrate feeds but were lower (*p* < 0.01) for the mash forms (UREA-M and ALKA-M) than the pelleted concentrate feeds. For the grass silages, the IVDMD significantly decreased with increasing stage of maturity (Ecut > Mcut > Lcut). For the mixed diets, there was no interaction effect of grass silage by concentrate feed type for IVDMD. However, both the effects of grass silage quality and concentrate feed type were significant (*p* < 0.01), reflecting the patterns as observed for the grass silages and concentrate feed types in their respective feed categories.

### 3.4. Ammonia Nitrogen and VFA Production

Data on in vitro fermentation products of the concentrate feeds and grass silages are provided in Table 3. For the concentrate feeds, there was no significant difference in pH_48_, total VFAs, and molar proportions (% of total VFAs) of valeriate, isovaleriate, and isobutyrate. However, there was a tendency for lower NH_3_-N and acetate-to-propionate ratio (Acet:Prop) in the pelleted concentrate feeds compared to mash forms. Furthermore, the molar proportions of acetate, propionate, and butyrate differed (*p* < 0.05) among the concentrate feeds. As a result, CONT-P had higher acetate and lower butyrate than the other three feeds. The UREA-M and ALKA-M produced higher butyrate levels, with ALKA-P producing an intermediate value.

For the grass silages, there was a strong influence of silage quality on the above parameters (*p* < 0.01) except for total VFAs and butyrate. As such, NH_3_-N, molar proportions of propionate, isobutyrate, valerate, and isovalerate decreased with increasing stage of maturity. Conversely, acetate, the ratio of acetate to propionate, and pH_48_ were increased with increasing stage of maturity (i.e., Lcut > Mcut > Ecut).

For the mixed feeds (provided in Table S1), in a similar fashion to the gas production, the effects of concentrate feeds and their interaction with the silage quality on fermentation parameters were not significant. Ammonia nitrogen, and molar proportions of propionate, butyrate, and branched-chain VFA decreased while molar proportions of acetate and pH_48_ increased in the order of increasing stage of maturity in the mixed diets. The total VFAs were not affected.

### 3.5. Estimated Utilizable Crude Protein and Energy Values

Estimated uCP and energy values (ME and NE_l_) of the concentrate feeds and grass silages are provided in Table 3. The uCP of ALKA-P was higher than that of CONT-P. The ALKA-M had the lowest uCP values compared to the other three. The estimated energy values of the UREA-M and ALKA-M were not different from the ALKA-P. However, CONT-P had a marginally lower (*p* = 0.041) ME value compared to the UREA-M and ALKA-M feeds. For the grass silages, the values for uCP, and estimated energy values decreased with increasing stage of maturity. For the mixed feeds, the interaction effects of concentrate feed type and grass silage quality were not significant, and the above parameters followed the quality of grass silages and/or concentrate feed type (Supplementary Materials, Table S1).

## 4. Discussion

The effects of concentrate feed formulations, contrasting grass silage qualities, and mixtures of these silages and concentrates assuming hypothetical dairy cow rations (i.e., 0.55:0.45; grass silage: concentrate, on a DM basis) were tested on in vitro gas production parameters and estimated utilizable crude protein and energy values. Even though noticeable differences in the estimated parameters were observed within concentrates and grass silages, the in vitro gas production data lacked any interaction effects between the concentrate feeds and grass silage qualities for the mixed diets. Therefore, the main effects of concentrate feeds and grass silage qualities are discussed hereunder with sparing details of the mixed diets. The observed patterns for tested parameters on the mixed diets are provided with the Supplementary Materials (Table S1, Figures S1–S4).

### 4.1. Gas Production

The four concentrate feeds displayed comparable values for gas volume at different time points, mean rate of substrate fermentation, tVFA, pH_48_ of the incubated medium, and the molar proportions of the minor VFAs (i.e., valeriate, isovaleriate, and isobutyrate). However, the observed differences in time taken to produce 50% of the asymptotic gas volume and the molar proportions of the main VFAs (i.e., acetate, propionate, and butyrate) between the pelleted (ALKA-P and CONT-P) and mash form (UREA-M and ALKA-M) diets suggested shifts in the in vitro substrate fermentation pattern. The shorter time taken to produce 50% of the asymptotic gas volume and higher molar proportion of propionate with the pelleted diets could be due to pelleting heat treatments and starch gelatinization in the process. It is known that starch gelatinization increases with pelleting heat treatments [36], with a subsequent increase in the rate of fermentation [37]. Unfortunately, it was not possible to estimate the rate of fermentation for specific nutrients from the in vitro work. However, dry sieving particle size distribution (Figure S3) showed that the pelleted diets had relatively greater portions of finer fractions (particle size ≲ 0.25 mm, as also indicated by the smaller geometric mean particle size in these feeds in Table 1) and lesser portions of large particles (size > 0.8 mm) than the mash forms for feed materials milled through the 1.0 mm sieve size. These different fractions may differ in chemical composition (e.g., high starch and CP but low NDF in the finer fractions as reported by Spanghero et al. [38]) with effects at least on early colonization of the feed materials by microbes and hence substrate fermentation [39,40]. This is further supported by the subtle but higher peaks of the substrate fermentation rate (Figure 1) along with the observed higher IVDMD of the pelleted diets relative to the mash forms in the absence of any noticeable difference in the gas volume and total VFAs among the concentrate feeds.

Gas production, fermentation products, and pH data on the grass silages reflected the contrasting silage qualities. Differences in the type of substrate available for fermentation—greater cell wall content along with decreasing contents of water-soluble carbohydrates and CP in the late cut grass silage—were expected to affect the pattern of fermentation and DM fermented [41,42]. Therefore, the observed decreasing gas volume, increasing proportions of acetate, and decreasing proportion of propionate in the tVFA with the increasing maturity of grass silages aligned with expectations, given the contrasting qualities of grass silages. Furthermore, proteolysis and the subsequent metabolism of the products of proteolysis by microorganisms are expected to increase the production of amino acid-derived metabolites such as branched-chain VFAs [43,44,45,46]. Thus, the decreasing level of branched short-chain VFAs with increasing stage of maturity of the grass silages observed is suggestive of the quantitatively reduced level of the breakdown of dietary proteins with the late cut grass silage.

In the mixed diets, the in vitro fermentation patterns and level of fermentation products largely mirrored the grass silage qualities. The lack of any interaction effects between concentrate feed type and grass silage quality in these diets could be argued as either an artifact of a batch culture system or a lack of any differential modulatory effects of the concentrate feeds on the grass silages at the forage-to-concentrate mixing ratio used here. The latter appears to be more likely here, even though feed interactions remain poorly understood and difficult to investigate [47]. Previous efforts to investigate such effects of varied concentrate feeds and inclusion levels with grass silages of contrasting qualities using in vitro fermentation showed modulatory effects of the concentrate feed inclusion level rather than concentrate feed type [48]. As such, mixing three concentrate feeds differing in protein source (i.e., soy-based, yeast-based, and barley-based) with two contrasting grass silage qualities (i.e., early vs. late cut) at varying forage-to-concentrate mixing ratios, the late cut grass silage gained more in IVDMD than the early cut silage when the concentrate feed inclusion level was at or above 50% in the mixed diets [48]. Similarly, with goats fed two different qualities of basal diets (i.e., grass hay vs. alfalfa hay) mixed with concentrate feed at 70/30 and 30/70 (F:C), interaction effects for the in vivo apparent digestibility values of nutrients were reported [49]. As a result, the low-quality grass hay (i.e., CP = 94.7 g/kg DM and NDF = 506 g/kg DM) produced higher in vivo digestibility than the alfalfa hay (CP = 192 g/kg DM, NDF = 428 g/kg DM) only at the higher concentrate feed inclusion level. Therefore, it is more likely that it would have required a higher level of concentrate inclusion for such an effect to occur in our study, but then such elevated concentrate levels would be of limited importance under field conditions.

### 4.2. Estimated Utilizable Crude Protein and Energy Values

The uCP is an estimate of the sum of microbial crude protein (MCP) and undegraded feed protein in the rumen (RUP) that enters the duodenum. For the concentrate feeds, the estimated uCP value of ALKA-P was higher than the other concentrate feeds. The lack of a technique for accurate separation of MCP from RUP in digesta limits any conclusive evidence [50] as to the source of increased uCP, especially under in vitro conditions. However, since the Home n’ Dry pellets contributed part of the CP of the ALKA-P as NPN compared to that of CONT-P, it could be argued here that the observed uCP is largely due to higher MCP rather than RUP. This is further supported by the IVDMD (discussed below) and also by the relatively lower acetate-to-propionate ratio in the VFAs of the feed, suggestive of the MCP efficiency [51]. Our data depicting the inverse relationship between estimated uCP and the acetate-to-propionate ratio in the incubated media (Figure S4) further support this view. Overall, the uCP values of the feeds investigated here are lower than other in vitro works containing comparable dietary CP levels [32,52,53]. The uCP level of a given diet declines with extended incubation duration [32,53,54] or with slower passage rate [32] due to reduced microbial efficiency [55,56]. Therefore, the relatively extended (i.e., 48 h) incubation here in contrast to other in vitro studies (i.e., 24 h) along with other sources of variation in the in vitro fermentation kinetics of feedstuffs [57] could explain the observed uCP values of our feeds. However, because of the high correlations between the uCP values estimated using different retention times, Gidlund et al. [53] suggested that this is not a major issue for ranking the feeds.

Estimated ME and NE_l_ values based on in vitro gas production data provided subtle but significant differences between the pelleted and mash form concentrate feeds. Given that the feeds had comparable gas volume at 24 h of incubation, the differences in energy values appear to be due to differences in the chemical composition of the feeds as the formula for estimating ME allocates fixed coefficients to individual chemical constituents (e.g., CP, CF, ash) [33]. The observed greater IVDMD value of the pelleted diets compared to the mash form diets in the absence of supporting data on cumulative gas production, mean rate of substrate fermentation, tVFA, and pH_48_ was not clear. However, it could be attributed to the differences in the fermentation pattern as indicated by the level of the molar proportions of specific VFAs, the ratio of acetate to propionate in the VFAs, and any differences in the microbial cell yield from organic matter fermentation. Microbial cell yield is not measured here, but it is said to be a variable factor with a direct effect on the amount of fermentation acids produced [58]. Given again comparable CP in the diets, the marginally lower ammonia nitrogen concentration, greater uCP and IVDMD of the pelleted diets (i.e., ALKA-P and CONT-P) relative to the mash diets (i.e., UREA-M and ALKA-M) would suggest increased microbial growth in the pelleted diets.

Differences in the pH of the original feed materials as influenced by the processing method (ALKA-P vs. UREA-M and ALKA-M) and ingredient composition (e.g., CONT-P vs. ALKA-P) were anticipated to result in differences in the in vitro pH_48_. However, the pH_48_ was not different among these feeds, aligning with the tVFA but against our hypothesis and the observed differences in the IVDMD. Even though feedstuffs are expected to vary considerably in their buffering capacity [16], at least under in vivo conditions it is stated that the direct buffering by diets is much less than buffering by saliva [58]. The in vitro rumen fermentation is a highly buffered system; for instance, we used two portions of buffer solution to one portion of rumen inoculum in our experiment. Therefore, this could noticeably mask the expected differences in the pH_48_ among the concentrate feeds. Whether in vivo experiments with dairy cows fed at comparable or higher levels of concentrate feed and grass silage mixtures will conform to the observed results is currently being studied, and yet to be seen.

For the grass silages, the greater pH_48_ observed with increasing stage of maturity could be due to the initial higher pH of the late cut grass silage along with the numerically lower VFAs produced after 48 h of incubation, and other non-volatile organic acids (not measured here) reported to affect the buffering capacity of feeds [59]. The estimated uCP and energy values for the silages aligned with expectations, implying that good quality (i.e., early cut) grass silage can markedly cover the protein and energy requirements of ruminants with minimal recourse to concentrates, as was also observed with dairy cows [60] and growing/finishing bulls [23].

## 5. Conclusions

The alkaline pelleted concentrate feed produced comparable in vitro degradation characteristics to that of the control concentrate feed formulated with imported protein ingredients for high-yielding dairy cows. This is supported by the observed fermentation products, estimated uCP, ME, NE_l_, and IVDMD values. As all the concentrate feeds imitated a well-buffered rumen condition, given the endpoint pH of the in vitro incubation_,_ and due to the adequately buffered nature of the in vitro system, it was inconclusive whether the ALKA-P would provide a higher buffering capacity than the CONT-P in vivo. The results on grass silages and their mixtures with concentrate feeds highlight the importance of grass silage quality—as modulated by cutting age—on the in vitro fermentation quality, estimated uCP, and ME values with implications for expected in vivo response in ruminants.

## Figures and Tables

**Table 1:** Chemical composition and related parameters of the feeds.

	Concentrates				Grass Silages		
Ingredients, g/kg	CONT-P	ALKA-P	UREA-M	ALKA-M	Ecut	Mcut	Lcut
Barley ^1^	383	667	637	667			
Crushed wheat	90.0	100	100	100			
Crushed oats	100	-	-	-			
Crushed corn	80.0	-	-	-			
Beet pulp	50.0	-	-	-			
Corn gluten meal	20.0	55.7	55.7	55.7			
Rapeseed expeller	40.0	64.3	79.9	64.3			
Soybean product ^2^	107	-	-	-			
Peas	20.0	-	-	-			
Feed-grade urea	-	-	12.6	-			
Molasses	60.0	60.0	60.0	60.0			
Others ^3^	50.0	53.0	54.4	53.0			
Composition, g/kg DM							
Crude protein	197	192	215	202	227	166	102
aNDFom	188	153	178	166	333	477	639
Ash	62.7	70.7	68.3	73.4	76.8	67.8	58.5
ADFom	70.5	53.5	54.8	56.1	189	269	359
Crude fat	30.9	43.4	36.8	36.5	41.4	35.4	32.9
Starch	447	414	445	463	na	na	na
WSC	na	na	na	na	138.2	110.3	82.3
RestCH0	74.2	127.8	57.6	59.0	182.8	142.8	85.4
pH and particle size							
pH ^4^	6.95	8.00	6.94	6.33	4.20	4.39	4.60
Particle size ^5^, μm	252	282	450	443			

Feeds: CONT-P = pelleted control concentrate feed, ALKA-P = pelleted alkaline concentrate feed, UREA-M = concentrate feed formulated with feed-grade urea in a mash form using ALKA-P ingredients, ALKA-M =ALKA-P in a mash form avoiding the ammoniation step, Ecut = early cut grass silage, Lcut = late cut grass silage, and Mcut = a mixture (1:1, on dry matter basis) of the Ecut and Lcut. ^1^ Barley in the ALKA-P contains 20% alkaline grain (named Alkagrain150), whereas in the ALKA-M it comes with barley blended directly with Home n’ Dry pellets; ^2^ the soybean product is a mixture of 9.3% soybean meal and 90.7% soypass (i.e., a lignosulfonate-treated soybean meal); ^3^ the sum of minor ingredients delivering micro- and macro-minerals; ^4^ pH of the feeds measured in triplicates according to Jasaitis et al. [16]; ^5^ geometric mean particle size of concentrates milled to pass through a 1.0 mm sieve size as used for in vitro incubation. Composition: aNDFom = Neutral detergent fiber assayed with heat-stable amylase and corrected for residual ash; ADFom = Acid detergent fiber corrected for residual ash; WSC = water-soluble carbohydrates; RestCHO = Residual carbohydrate for concentrate feeds calculated as 1000—(aNDFom + CP + Starch + Ash + Crude fat), whereas for grass silages it includes silage fermentation products; na = not analyzed.

**Table 2:** Gas production kinetics and parameter estimates of the concentrate feeds and grass silages.

Treatments	GP_12_	GP_24_	GP_48_	A	B	C	R_gp_
Concentrate feeds							
CONT-P	188.5	243.0	270.0	280.5	8.10 ^b^	1.76	0.066
ALKA-P	183.3	239.0	263.7	272.9	8.40 ^b^	1.90	0.070
UREA-M	178.6	244.0	271.9	268.2	9.40 ^a^	1.89	0.066
ALKA-M	189.5	253.2	285.8	288.6	9.10 ^a^	1.89	0.066
SE	4.84	5.75	9.05	6.22	0.18	0.07	0.003
*p*-value	0.39	0.39	0.39	0.16	<0.001	0.52	0.75
Grass silages							
Ecut	184 ^a^	239 ^a^	262 ^a^	275 ^a^	8.00 ^c^	1.75 ^a^	0.066 ^a^
Mcut	153 ^b^	205 ^b^	232 ^b^	250 ^b^	9.20 ^b^	1.58 ^b^	0.056 ^b^
Lcut	120 ^c^	179 ^c^	217 ^b^	237 ^c^	12.7 ^a^	1.46 ^c^	0.044 ^c^
SE	4.29	5.81	6.23	2.84	0.26	0.03	0.002
*p*-value	<0.001	<0.001	0.001	<0.001	<0.001	<0.001	<0.001

GP12, GP24, and GP48 are net gas volume (mL/g DM) at 12, 24, and 48 h of fermentation, respectively. A, B, and C are parameter estimates of asymptotic gas volume (mL/g DM), time to produce 50% asymptotic gas volume (h), and shape parameter characteristic of individual curve, respectively, whereas R_gp_ is average substrate fermentation rate (/h). Concentrate feeds: CONT-P = pelleted control concentrate feed, ALKA-P = pelleted alkaline concentrate feed, UREA-M = concentrate feed formulated with feed-grade urea in a mash form using ALKA-P ingredients, ALKA-M = ALKA-P in a mash form avoiding the ammoniation step. Grass silages: Ecut = early cut, Lcut = late cut, Mcut = a mixture (1:1, on a dry matter basis) of the Ecut and Lcut. ^a,b,c^ Least square means in column within each feed category carrying different superscripts are significantly different from each other at *p* < 0.05.

**Table 3:** In vitro rumen fermentation products, utilizable CP (uCP, g/kg DM), in vitro DM digestibility (IVDMD, %), and energy values (MJ/kg DM) of concentrate feeds and grass silages after 48 h of fermentation.

	Concentrate Feeds						Grass Silages				
	CONT-P	ALKA-P	UREA-M	ALKA-M	SE	*p*-Value	Ecut	Mcut	Lcut	SE	*p*-Value
Fermentation products											
NH_3_-N	354	334	396	388	14.3	0.055	409 ^a^	335 ^b^	284 ^c^	5.51	<0.001
Acet	63.2 ^a^	61.4 ^b^	61.1 ^b^	61.1 ^b^	0.37	0.014	61.4 ^b^	64.9 ^a^	66.2 ^a^	0.4	<0.001
Prop	18.0 ^a^	18.2 ^a^	16.1 ^b^	16.1 ^b^	0.42	0.012	21.8 ^a^	20.6 ^b^	19.4 ^c^	0.55	0.006
But	14.4 ^c^	15.9 ^b^	18.5 ^a^	18.5 ^a^	0.37	<0.001	11.8	10.5	10.9	0.33	0.08
Isobut	1.08	1.03	1.03	1.05	0.02	0.36	1.19 ^a^	1.05 ^b^	0.91 ^c^	0.04	0.02
Val	1.43	1.5	1.53	1.54	0.03	0.15	1.72 ^a^	1.33 ^b^	1.08 ^c^	0.02	<0.001
Isoval	1.86	1.77	1.77	1.82	0.05	0.55	2.09 ^a^	1.68 ^b^	1.54 ^c^	0.05	0.002
tVFA	96.9	94.7	97.3	97	3.84	0.96	100	103	90.5	3.54	0.12
Ace:Pro	3.50	3.39	3.84	3.83	0.126	0.089	2.82 ^c^	3.14 ^b^	3.41 ^a^	0.054	0.002
pH_48_	6.41	6.41	6.41	6.41	0.013	0.98	6.30 ^c^	6.37 ^b^	6.41 ^a^	0.009	<0.001
Dry matter digestibility and estimated feeding values											
IVDMD	85.9 ^a^	84.4 ^a^	76.3 ^b^	78.4 ^b^	1.6	0.002	77.1 ^a^	68.5 ^b^	50.0 ^c^	1.75	<0.001
uCP	124.3 ^b^	131.5 ^a^	124.5 ^b^	117.4 ^c^	1.55	0.003	120.3 ^a^	105.5 ^b^	75.7 ^c^	2.57	<0.001
ME	12.4 ^b^	12.6 ^ab^	12.7 ^a^	12.7 ^a^	0.09	0.041	11.6 ^a^	10.0 ^b^	8.5 ^c^	0.09	<0.001
NE_L_	7.7 ^b^	7.9 ^a^	8.0 ^a^	8.0 ^a^	0.07	0.027	7.1 ^a^	6.0 ^b^	4.9 ^c^	0.06	<0.001

NH_3_-N (mg/L) = ammonia nitrogen; tVFA (mmol/L) = total short-chain volatile fatty acids; Acet, Prop, But, Val, Isobut, and Isoval are acetate, propionate, butyrate, valeriate, isobutyrate, and isovaleriate, respectively, as % of tVFA; Acet:Prop is the ratio of acetate to propionate; pH_48_ = pH of the incubated medium after 48 h. Concentrate feeds: CONT-P = pelleted control concentrate feed, ALKA-P = pelleted alkaline concentrate feed, UREA-M = concentrate feed formulated with feed-grade urea in a mash form using ALKA-P ingredients, ALKA-M =ALKA-P in a mash form avoiding the ammoniation step. Grass silages: Ecut = early cut grass silage, Lcut = late cut grass silage, Mcut = a mixture (1:1, on a dry matter basis) of the Ecut and Lcut. ^a,b,c^ Means within a row for each feed category carrying different superscripts are significantly different from each other at *p* < 0.05.

## Data Availability

Data can be obtained upon request from the corresponding author.

## Supplementary Materials

The following supporting information can be downloaded at: https://www.mdpi.com/article/10.3390/ani13172695/s1, Table S1. In vitro rumen fermentation characteristics of mixtures of four concentrate feeds and three contrasting grass silage qualities; Figure S1. In vitro gas production characteristics of mixtures of four concentrate feeds and three contrasting grass silage qualities; Figure S2. In vitro dry matter digestibility of mixtures of four concentrate feeds and three contrasting grass silage qualities after 48 h of fermentation; Figure S3. Particle size distribution of the four concentrate feeds milled to pass through a 1.0 mm sieve size presented as mass fraction retained on different sieve sizes using the dry sieving method; and Figure S4. The relationship between estimated utilizable crude protein value and the ratio of acetate to propionate in the incubated media of concentrate feeds, grass silages, and their mixtures.

## References

[1] Nafstad O., Nesse K.A., Alvseike O.A., Kjos A.-K., Nafstad O., Odden H., Arne T.R., Saltnes T., Ytterdahl M. (2017). Økt matproduksjon på norske ressurser-kan vi ikke bare dyrke mer mat da?. Kjøttets Tilstand: Status i Norsk Kjøtt- og Eggproduksjon.

[2] Waalen W.M. Grain and Forage Seed Crops. https://nibio.no/en/subjects/food/grain-and-forage-seed-crops.

[3] The Norwegian Food Safety Authority Norway 2015. https://www.mattilsynet.no/om_mattilsynet/annual_report_the_norwegian_food_safety_authority_2015.22993.

[4] Home n’ Dry pellets. https://www.homendry.com/alkagrain.

[5] Humer E., Zebeli Q. (2017). Grains in ruminant feeding and potentials to enhance their nutritive and health value by chemical processing. Anim. Feed Sci. Technol..

[6] Li M.M., White R.R., Guan L.L., Harthan L., Hanigan M.D. (2021). Metatranscriptomic analyses reveal ruminal pH regulates fiber degradation and fermentation by shifting the microbial community and gene expression of carbohydrate-active enzymes. Anim. Microbiome.

[7] Mathison G.W. (1996). Effects of processing on the utilization of grain by cattle. Anim. Feed Sci. Technol..

[8] Owens F.N., Secrist D.S., Hill W.J., Gill D.R. (1997). The effect of grain source and grain processing on performance of feedlot cattle: A review. J. Anim. Sci..

[9] Bertipaglia L.M.A., Fondevila M., van Laar H., Castrillo C. (2010). Effect of pelleting and pellet size of a concentrate for intensively reared beef cattle on in vitro fermentation by two different approaches. Anim. Feed Sci. Technol..

[10] Wan Y., Ma R., Khalid A., Chai L., Qi R., Liu W., Li J., Li Y., Zhan K. (2021). Effect of the Pellet and Mash Feed Forms on the Productive Performance, Egg Quality, Nutrient Metabolism, and Intestinal Morphology of Two Laying Hen Breeds. Animals.

[11] Broderick G.A., Craig W.M. (1980). Effect of heat treatment on ruminal degradation and escape, and intestinal digestibility of cottonseed meal protein. J. Nutr..

[12] Lund P., Weisbjerg M.R., Kristensen T. (2004). The effect of heat treatment on degradability and microbial synthesis of protein in the rumen. J. Anim. Feed Sci..

[13] Mayne C.S. (1990). An evaluation of an inoculant of Lactobacillus plantarum as an additive for grass silage for dairy cattle. Anim. Prod..

[14] Bakken A.K., Vaga M., Hetta M., Randby Å.T., Steinshamn H. (2017). Protein characteristics in grass–clover silages according to wilting rate and fermentation pattern. Grass Forage Sci..

[15] Dawson L.E.R., Kirkland R.M., Ferris C.P., Steen R.W.J., Kilpatrick D.J., Gordon F.J. (2002). The effect of stage of perennial ryegrass maturity at harvesting, fermentation characteristics and concentrate supplementation, on the quality and intake of grass silage by beef cattle. Grass Forage Sci..

[16] Jasaitis D.K., Wohlt J.E., Evans J.L. (1987). Influence of Feed Ion Content on Buffering Capacity of Ruminant Feedstuffs In Vitro. J. Dairy Sci..

[17] (1999). Animal Feeding Stuffs—Determination of Moisture and Other Volatile Matter Content.

[18] (2002). Animal Feeding Stuff—Determination of Crude Ash.

[19] AOAC International (2002). Official Methods of Analysis. Association of Official Analytical Chemists (Method 2001.11).

[20] Thiex N.J., Manson H., Anderson S., Persson J.Å. (2002). Determination of crude protein in animal feed, forage, grain, and oilseeds by using block digestion with a copper catalyst and steam distillation into boric acid: Collaborative study. J. AOAC Int..

[21] AACC (2000). Method 76-13.01—Total starch assay procedure (Megaenzyne amyloglucosidase/alpha-amylase method). Approved Methods of Analysis.

[22] Horwitz W., AOAC International (Association of Official Analytical Chemists) (2000). Official Method 973.18 Fiber (acid detergent) and lignin in animal feed. Official Methods of Analysis of AOAC International.

[23] Randby Å.T., Nørgaard P., Weisbjerg M.R. (2010). Effect of increasing plant maturity in timothy-dominated grass silage on the performance of growing/finishing Norwegian Red bulls. Grass Forage Sci..

[24] Volden H., Volden H. (2011). Feed calculations in NorFor. NorFor-The Nordic Feed Evaluation System.

[25] European Commission (2009). Commission Regulation. (No 152/2009. 27 January 2009). Laying Down the Methods of Sampling and Analysis for the Official Control of Feed. Determination of Crude Oils and Fats.

[26] Randall E.L. (1974). Improved method for fat and oil analysis by a new process of extraction. J. AOAC.

[27] ASAE (1997). Method of Determining and Expressing Fineness of Feed Materials by Sieving. ASAE Standard S319.2. Agricultural Engineers Yearbook of Standards.

[28] Kidane A., Overland M., Mydland L.T., Prestlokken E. (2018). Interaction between feed use efficiency and level of dietary crude protein on enteric methane emission and apparent nitrogen use efficiency with Norwegian Red dairy cows. J. Anim. Sci..

[29] Goering H.K., Van Soest P.J. (1970). Forage Fiber Analysis (Apparatus, Reagents, Procedures and Some Applications).

[30] Volden H., NorFor (2011). NorFor—The Nordic Feed Evaluation System.

[31] Alvarez C., Andersen T.O., Eikanger K.S., Hamfjord I.W., Niu P., Weiby K.V., Årvik L., Dörsch P., Hagen L.H., Pope P.B. (2022). Methane inhibition by Asparagopsis taxiformis with rumen fluid collected from ventral and central location-a pilot study. Acta Agric. Scand. A Anim. Sci..

[32] Edmunds B., Südekum K.H., Spiekers H., Schuster M., Schwarz F.J. (2012). Estimating utilisable crude protein at the duodenum, a precursor to metabolisable protein for ruminants, from forages using a modified gas test. Anim. Feed Sci. Technol..

[33] (2008). GfE New equations for predicting metabolisable energy of grass and maize products for ruminants. Proc. Soc. Nutr. Physiol..

[34] Weißbach F., Schmidt L., Kuhla S. (1996). Vereinfachtes verfahren zur berechnung der NEL aus der umsetzbaren energie. Proc. Soc. Nutr. Physiol..

[35] Groot J.C.J., Cone J.W., Williams B.A., Debersaques F.M.A., Lantinga E.A. (1996). Multiphasic analysis of gas production kinetics for in vitro fermentation of ruminant feeds. Anim. Feed Sci. Technol..

[36] Truelock C.N., Tokach M.D., Stark C.R., Paulk C.B. (2020). Pelleting and starch characteristics of diets containing different corn varieties. Transl. Anim. Sci..

[37] Holm J., Lundquist I., Björck I., Eliasson A.C., Asp N.G. (1988). Degree of starch gelatinization, digestion rate of starch in vitro, and metabolic response in rats. Am. J. Clin. Nutr..

[38] Spanghero M., Magni G., Boselli E., Piombino M., Mason F., Cozzi G. (2017). Prediction of metabolisable energy content of commercial total mixed rations (TMR) for lactating dairy cows based on gas production measured into two TMR fractions. Anim. Feed Sci. Technol..

[39] Gerson T., King A.S.D., Kelly K.E., Kelly W.J. (1988). Influence of particle size and surface area on in vitro rates of gas production, lipolysis of triacylglycerol and hydrogenation of linoleic acid by sheep rumen digesta or Ruminococcus flavefaciens. J. Agric. Sci..

[40] Offner A., Bach A., Sauvant D. (2003). Quantitative review of in situ starch degradation in the rumen. Anim. Feed Sci. Technol..

[41] Beuvink J.M.W., Spoelstra S.F. (1992). Interactions between substrate, fermentation end-products, buffering systems and gas production upon fermentation of different carbohydrates by mixed rumen microorganisms in vitro. Appl. Microbiol. Biotechnol..

[42] John A., Barnett G., Reid R.L. (1957). Studies on the production of volatile fatty acids from grass by rumen liquor in an artificial rumen: II. The volatile fatty acid production from dried grass. J. Agric. Sci..

[43] Liu X., Blouin J.-M., Santacruz A., Lan A., Andriamihaja M., Wilkanowicz S., Benetti P.-H., Tomé D., Sanz Y., Blachier F. (2014). High-protein diet modifies colonic microbiota and luminal environment but not colonocyte metabolism in the rat model: The increased luminal bulk connection. Am. J. Physiol. Gastrointest. Liver Physiol..

[44] Ríos-Covián D., Ruas-Madiedo P., Margolles A., Gueimonde M., de Los Reyes-Gavilán C.G., Salazar N. (2016). Intestinal Short Chain Fatty Acids and their Link with Diet and Human Health. Front. Microbiol..

[45] Rasmussen H.S., Holtug K., Mortensen P.B. (1988). Degradation of Amino Acids to Short-Chain Fatty Acids in Humans: An in Vitro Study. Scand. J. Gastroenterol..

[46] Andries J.I., Buysse F.X., De Brabander D.L., Cottyn B.G. (1987). Isoacids in ruminant nutrition: Their role in ruminal and intermediary metabolism and possible influences on performances—A review. Anim. Feed Sci. Technol..

[47] Wood C.D., Manyuchi B. (1997). Use of an in vitro gas production method to investigate interactions between veld hay and Napier hay or groundnut hay supplements. Anim. Feed Sci. Technol..

[48] Kidane A., Hoøen A.J.H., Prestlokken E., Vhile S.G., Jensen R.B., Mydland L.T., Virkajärvi P., Hakala K., Hakojärvi M., Helin J., Herzon I., Jokela V., Peltonen S., Rinne M., Seppänen M., Uusi-Kämppä J. (2020). Interactive effects of three different compound feeds and two contrasting grass silages mixed at different proportions on in vitro gas production and fermentation kinetics. European Grassland Federation: 2020, Proceedings of the Organizing Committee of the 28th General Meeting of the Eurpean Grassland Federation, Online, 19–21 October 2020.

[49] Cantalapiedra-Hijar G., Yaáñ;ez-Ruiz D.R., Martín-García A.I., Molina-Alcaide E. (2009). Effects of forage:concentrate ratio and forage type on apparent digestibility, ruminal fermentation, and microbial growth in goats. J. Anim. Sci..

[50] Ørskov E.R., Fraser C., McDonald I. (1971). Digestion of concentrates in sheep: 3. Effects of rumen fermentation of barley and maize diets on protein digestion. Brit. J. Nutr..

[51] Chen Y.-H., Chen C.-Y., Wang H.-T. (2022). The Effect of Forage Source and Concentrated Liquid Feedstuff Supplementation on Improving the Synchronization of Ruminant Dietary Energy and Nitrogen Release In Vitro. Fermentation.

[52] Ramin M., Franco M., Roleda M.Y., Aasen I.M., Hetta M., Steinshamn H. (2019). In vitro evaluation of utilisable crude protein and methane production for a diet in which grass silage was replaced by different levels and fractions of extracted seaweed proteins. Anim. Feed Sci. Technol..

[53] Gidlund H., Vaga M., Ahvenjärvi S., Rinne M., Ramin M., Huhtanen P. (2018). Predicting omasal flow of nonammonia N and milk protein yield from in vitro-determined utilizable crude protein at the duodenum. J. Dairy Sci..

[54] Südekum K.H., Böttger C. The Hohenheim gas test for evaluating protein to ruminants. Proceedings of the 10th Nordic Feed Science Conference.

[55] Firkins J.L. (1996). Maximizing microbial protein synthesis in the rumen. J. Nutr..

[56] Hackmann T.J., Firkins J.L. (2015). Maximizing efficiency of rumen microbial protein production. Front. Microbiol..

[57] Keim J.P., Alvarado-Gilis C., Arias R.A., Gandarillas M., Cabanilla J. (2017). Evaluation of sources of variation on in vitro fermentation kinetics of feedstuffs in a gas production system. Anim. Sci. J..

[58] Allen M.S. (1997). Relationship between fermentation acid production in the rumen and the requirement for physically effective fiber. J. Dairy Sci..

[59] Muck R.E., Wilson R.K., O’Kiely P. (1991). Organic acid content of permanent pasture grasses. Irish J. Agric. Res..

[60] Álvarez C., Nielsen N.I., Weisbjerg M.R., Volden H., Eknæs M., Prestløkken E. (2022). High-digestible silages allow low concentrate supply without affecting milk production or methane emissions. J. Dairy Sci..

