# Supplementary material for: In Vitro Rumen Fermentation Characteristics, Estimated Utilizable Crude Protein and Metabolizable Energy Values of Grass Silages, Concentrate Feeds and Their Mixtures
Source: Animals (Basel). 2023 Aug 23;13(17):2695. doi: 10.3390/ani13172695 (PMC10486919; doi:10.3390/ani13172695)
Supplement: Supplementary file 1 [file animals-13-02695-s001.zip › animals-2530500-supplementary.pdf]

Supplementary Material for: In vitro rumen fermentation characteristics, estimated utilizable crude protein and metabolizable energy values of grass silages, concentrate feeds and their mixtures

Table S1. In vitro rumen fermentation characteristics of the mixtures of the four concentrate feeds and three contrasting grass silage qualities.

		In vitro gas production (mL/g DM)		In vitro fermentation products (% of tVFA, unless otherwise given)					*Estimated values per kg DM*	
Concentrate	*Grass silage*	GP_{12}	GP_{24}	NH_3-N (mg/L)	tVFA (mmol/L)	Acet	Prop	But	*ME (MJ)*	*uCP (g)*
CONT-P	Ecut	188.8	240.9	397.6	90.7	63.3	19.5	12.5	12.0	112.1
	Mcut	172.7	225.2	347.7	99.5	64.6	19.3	12.0	11.2	113.0
	Lcut	150.9	206.4	308.1	93.5	65.8	18.7	11.8	10.4	105.4
ALKA-P	Ecut	186.7	238.5	379.2	103.9	63.8	19.3	12.6	12.1	121.4
	Mcut	177.5	229.1	341.3	103.5	65.1	18.7	12.3	11.4	114.6
	Lcut	146.9	200.2	297.7	103.6	66.7	18.2	11.8	10.4	109.6
UREA-M	Ecut	186.0	240.5	411.6	107.4	63.2	18.1	14.4	12.1	115.2
	Mcut	173.4	225.9	361.9	101.9	64.2	17.7	14.1	11.4	107.3
	Lcut	151.7	206.2	316.2	94.5	65.2	17.4	13.8	10.5	103.8
ALKA-M	Ecut	188.2	242.7	391.4	105.1	62.7	18.6	14.3	12.1	113.0
	Mcut	170.3	224.2	352.6	96.6	63.8	18.1	14.1	11.3	106.4
	Lcut	148.0	203.8	314.7	93.2	65.1	17.5	13.7	10.4	98.0
	SE	3.70	4.34	8.26	6.53	0.63	0.35	0.43	0.07	3.36
Effects of :	Silage	<0.0001	<0.0001	<0.0001	0.348	<0.0001	<0.0001	0.004	0.010	0.039
	Concentrate	0.898	0.933	0.024	0.235	0.191	0.002	0.017	<0.0001	<0.0001
	Silage × Conc.	0.489	0.800	0.914	0.641	0.983	0.939	0.998	0.773	0.730

GP12 and GP24 are cumulative gas volumes at 12 and 24 h of incubation. Acet=Acetate, Prop= Propinate, But= Butyrate. Concentrate feeds: CONT-P = pelleted control concentrate feed, ALKA-P = pelleted alkaline concentrate feed, UREA-M = concentrate feed formulated with feed-grade urea in a mash form using ALKA-P ingredients, ALKA-M =ALKA-P in a mash form avoiding the ammoniation step. Grass silages: Ecut = early cut grass silage, Lcut = late cut grass silage, Mcut = a mixture (1:1, on a dry matter basis) of the Ecut and Lcut.

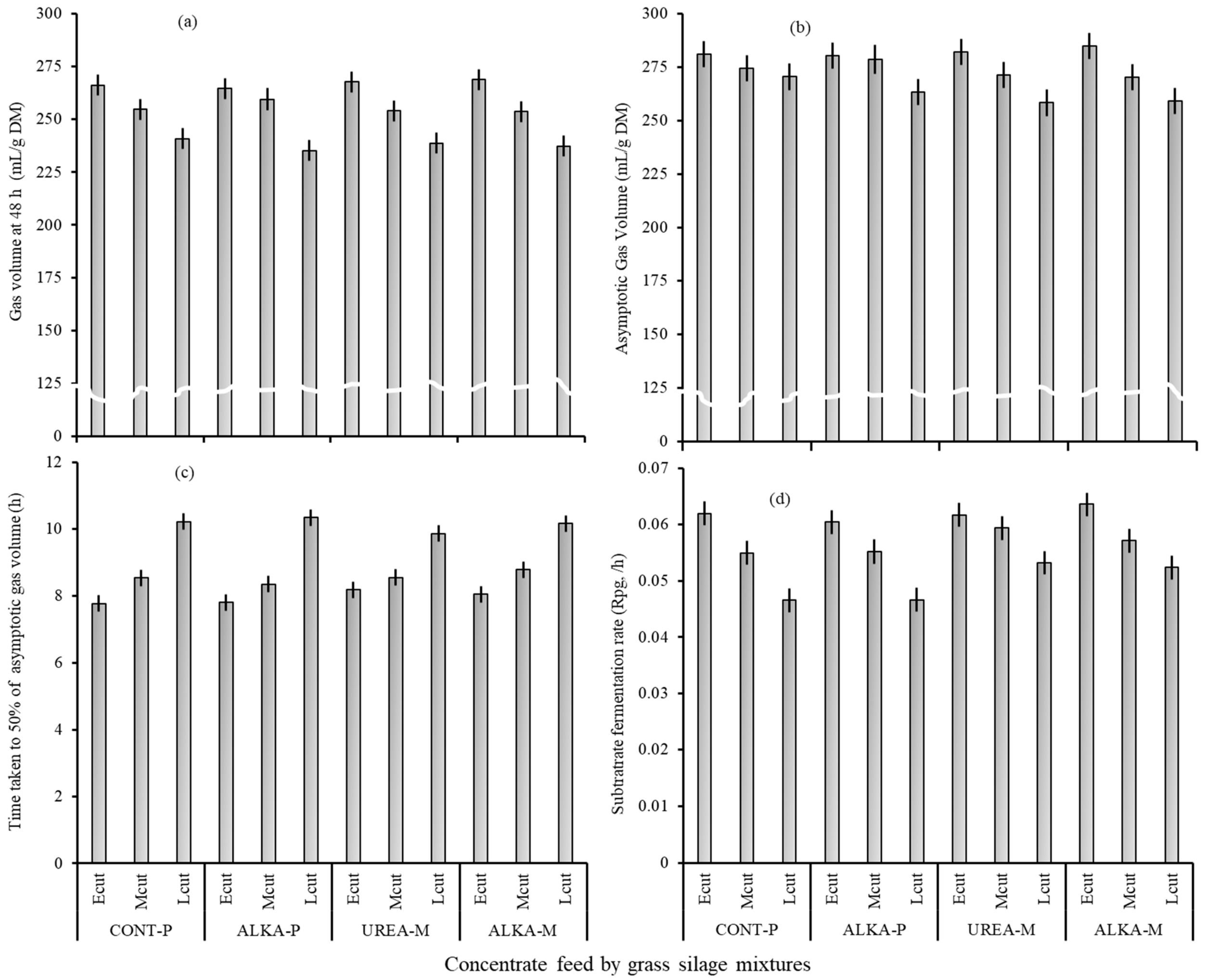

Figure S1. In vitro gas production characteristic mixtures of the four concentrate feeds and three contrasting grass silage qualities (means ± SEM). Panels: (**a**) achieved gas volume after 48 h of in vitro incubation, (**b**) estimated asymptotic gas volume, (**c**) time taken to produce 50% of the asymptotic gas volume, and (**d**) mean substrate fermentation rate. Concentrate feeds: CONT-P = pelleted control concentrate feed, ALKA-P = pelleted alkaline concentrate feed, UREA-M = concentrate feed formulated with feed-grade urea in a mash form using ALKA-P ingredients, ALKA-M =ALKA-P in a mash form avoiding the ammoniation step. Grass silages: Ecut = early cut, Lcut = late cut, Mcut = a mixture (1:1, on a dry matter basis) of the Ecut and Lcut.

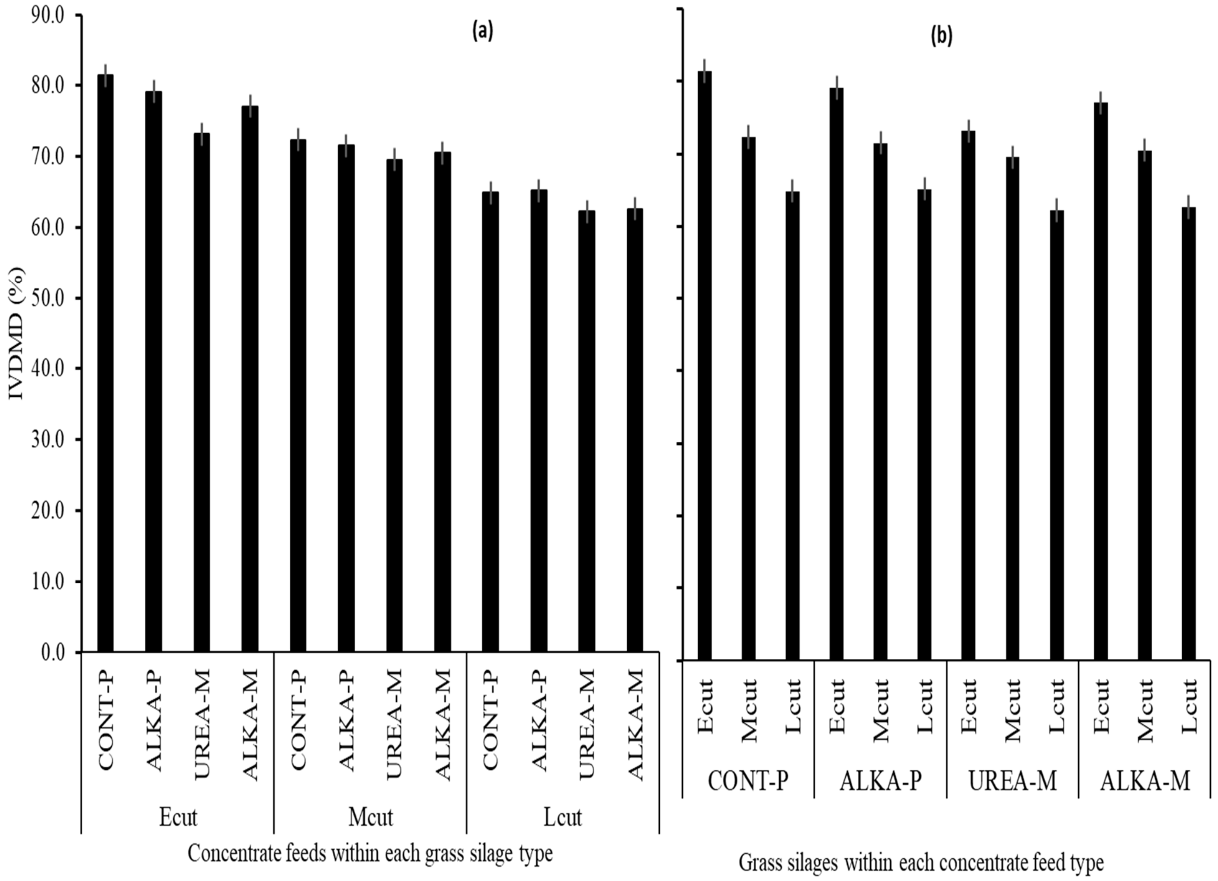

Figure S2. In vitro dry matter digestibility (IVDMD, % ± SEM) of mixtures of the four concentrate feeds and three contrasting grass silage qualities after 48 h of fermentation. Panels: (**a**) effects of concentrate feeds within each grass silage quality, (**b**) effects of grass silage quality within each concentrate feed type. Concentrate feeds: CONT-P = pelleted control concentrate feed, ALKA-P = pelleted alkaline concentrate feed, UREA-M = concentrate feed formulated with feed-grade urea in a mash form using ALKA-P ingredients, ALKA-M =ALKA-P in a mash form avoiding the ammoniation step. Grass silages: Ecut = early cut grass silage, Lcut = late cut grass silage, Mcut = a mixture (1:1, on a dry matter basis) of the Ecut and Lcut.

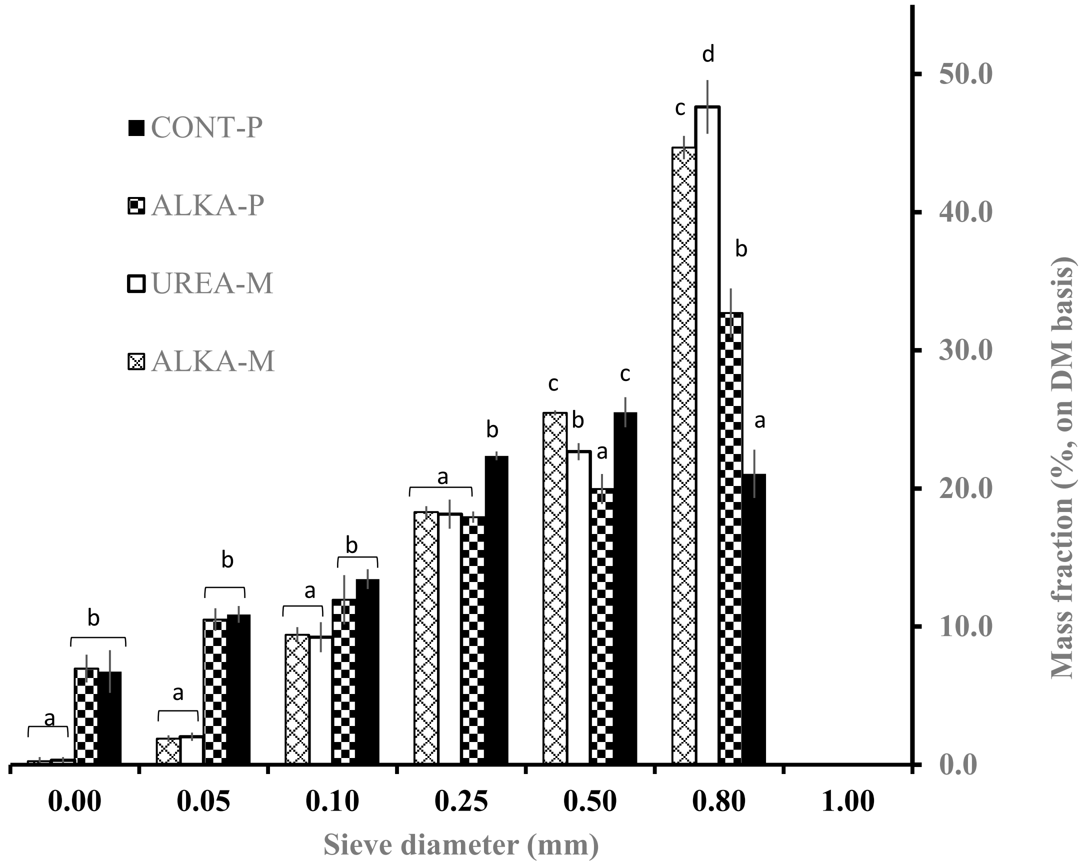

Figure S3. Particle size distribution of the four concentrate feeds milled to pass through a 1.0 mm sieve size presented as mass fraction (%, on a DM basis) retained on different sieve sizes using the dry sieving method. Concentrate feeds: CONT-P = pelleted control concentrate feed, ALKA-P = pelleted alkaline concentrate feed, UREA-M = concentrate feed formulated with feed-grade urea in a mash form using ALKA-P ingredients, ALKA-M =ALKA-P in a mash form avoiding the ammoniation step. Different superscripts on bars within a sieve diameter class stand for significantly different means at $p < 0.05$.

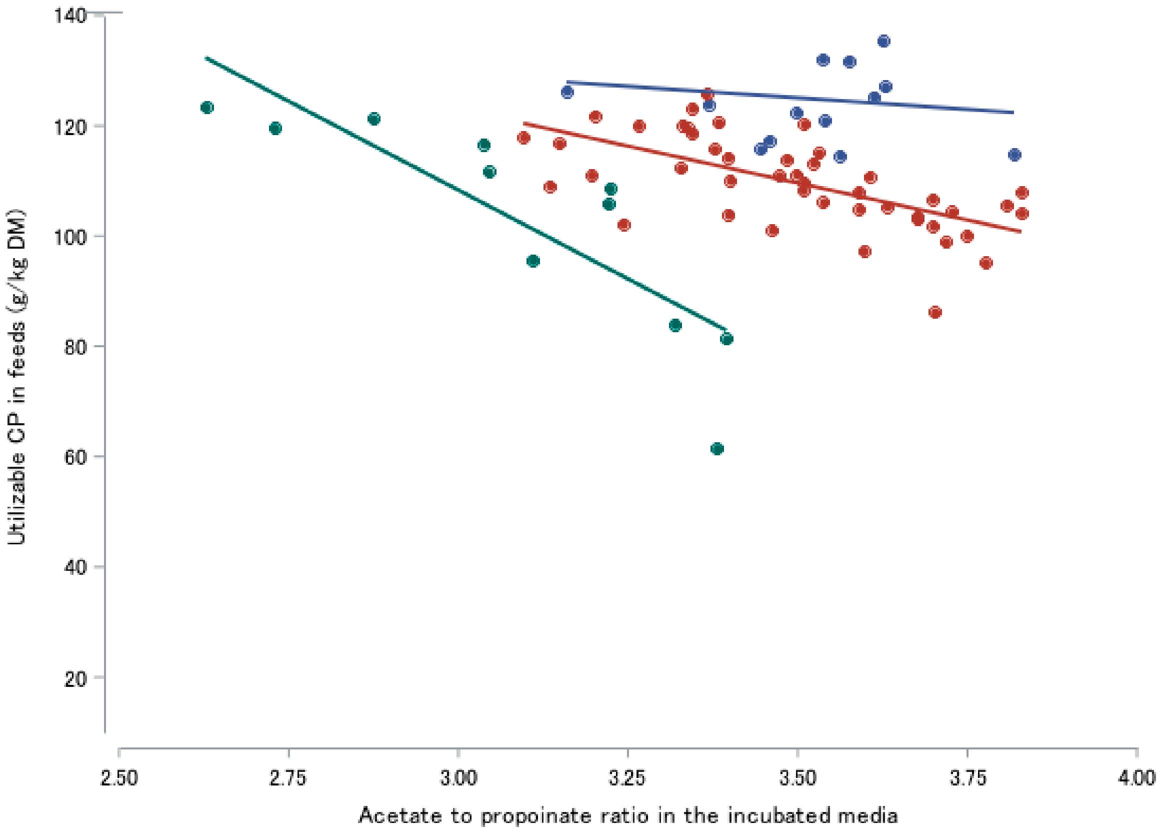

Figure S4. The relationship between estimated utilizable crude protein value and the ratio of acetate to propionate in the incubated media (Blue dots = concentrate feeds; Green dots = grass silages; Red dots= Mixed diets (0.55:0.45 F:C) of concentrate feeds, grass silages, and their mixtures. The respective regression lines within each feed category indicate response in estimated utilizable crude protein to an increasing acetate-to-propionate ratio from in vitro after 48 h incubation).
